# Synthesis of Polydopamine Functionalized Reduced Graphene Oxide-Palladium Nanocomposite for Laccase Based Biosensor

**DOI:** 10.1155/2016/5360361

**Published:** 2016-07-05

**Authors:** Da-Wei Li, Lei Luo, Peng-Fei Lv, Qing-Qing Wang, Ke-Yu Lu, An-Fang Wei, Qu-Fu Wei

**Affiliations:** ^1^Key Laboratory of Eco-Textiles, Ministry of Education, Jiangnan University, Wuxi, Jiangsu 214122, China; ^2^State Key Laboratory of Food Science and Technology, Wuxi 214122, China; ^3^Key Laboratory of Textile Fabric, Anhui Polytechnic University, Wuhu, Anhui 241000, China

## Abstract

Graphene based 2D nanomaterials have attracted increasing attention in biosensing application due to the outstanding physicochemical properties of graphene. In this work, palladium nanoparticles (Pd) loaded reduced graphene oxide (rGO) hybrid (rGO-Pd) was synthesized through a facile method. Laccase (Lac) was immobilized on rGO-Pd by utilizing the self-polymerization of dopamine, which generated polydopamine (PDA). The PDA-Lac-rGO-Pd nanocomposites were further modified on electrode surface to construct novel biosensing platform. The obtained electrochemical biosensor was applied in the detection of catechol, achieving excellent analytic results. Under the optimum condition, this biosensor possessed a linear range from 0.1 *µ*M to 263 *µ*M for catechol detection, the sensitivity reached 18.4 *µ*A mM^−1^, and the detection limit was as low as 0.03 *µ*M. In addition, the biosensor also showed good repeatability, reproducibility, anti-interference, and stability. Moreover, the novel Lac based biosensor was successfully used in the trace detection of catechol existing in real water environment.

## 1. Introduction

To our knowledge, phenolic compounds are hazardous and can cause severe health problems to human beings and animals [1]. These harmful compounds, as common industrial raw materials, widely exist in many industries and agriculture [1]. Catechol, as one of numerous phenolic contaminants, is a pivotal industrial chemical agent and can often be found in common foods such as crude beet sugar, coffee, and onion [2]. So, it has attracted a great deal of attention to exploit high-efficient detection methods toward catechol with low detection limit and high sensitivity. Up to now, a variety of catechol detection methods have been invented and applied, like gas chromatography, liquid chromatography, enzyme immunoassays, and so forth [3, 4]. These methods are restricted by their high cost, complicated analytic process, and fixed space location caused by the ponderous analysis instruments. Laccase (Lac) can catalyze the oxidation of phenols, coupled with reduction of O_2_ to H_2_O. On basis of this, a large number of Lac based biosensors have been prepared for the detection of catechol [5–8]. Laccase (Lac) based electrochemical biosensors offer a facile, simple, high-efficient analysis method for the detection of catechol, which is not affected by solution color and can achieve the microminiaturization of analysis apparatus.

Metal nanoparticles have been widely applied in both chemical sensors and biosensors due to their high surface area, high mechanical strength, ultralight weight, rich electronic properties, and excellent chemical and thermal stability [9, 10]. However, abundant metal nanoparticles often aggregate in the fabrication process of biosensor, which extremely affects their electrocatalysis toward substrates. As a result, combination of metal nanoparticles and other conductive materials becomes an effective way to solve the above problem. Graphene was discovered in 2004, which opened a new avenue to electrochemical biosensor research [11]. The unique properties of graphene (high thermal conductivity, fast electron transportation, excellent mechanical flexibility, and biocompatibility) make it become potential application material in electrochemical sensor [12, 13]. Palladium (Pd) nanoparticles loaded graphene oxide (GO) or reduced graphene oxide (rGO), combining their advantages together, have been applied in electrochemical enzyme-free sensors and electrochemical biosensors [14–16]. Dopamine (DA), existing in the brains and bodies of animals, is an important neurotransmitter of redox activity. Self-polymerization of DA generates polydopamine (PDA) with good biocompatibility, hydrophilicity, and excellent adhesiveness. Considering these merits of PDA, it has been applied in biosensing and multifunctional coating [17–21].

In this work, Pd nanoparticles loaded rGO (rGO-Pd) was synthesized through a facile method. Lac was immobilized on rGO-Pd by utilizing one-pot Lac-catalyzed oxidation of DA in an aqueous suspension containing Lac, rGO-Pd, and DA. The as-prepared PDA-Lac-rGO-Pd composite was used to modify glass carbon electrode (GCE) to construct novel PDA-Lac-rGO-Pd/GCE biosensor. The biosensor was employed to detect harmful phenolic compound (catechol) in water environment and achieved satisfactory analysis results. This study demonstrates that the PDA-Lac-rGO-Pd/GCE biosensor can serve as an effective water environmental monitor and may find potential applications in other research fields, like biofuel cell, biocatalyst, and so forth.

## 2. Materials and Methods

### 2.1. Reagents and Materials

Palladium acetate, expanded graphite powder, dopamine (DA), and catechol were purchased from Aladdin Chemical Reagent Co., Ltd. (Shanghai, China). Laccase (Lac, from Trametes, activity ≥ 10 U/mg) was obtained from Sigma-Aldrich and used without further purification. Other chemicals were all purchased from the Sinopharm Group Chemical Reagent Co., Ltd. (Shanghai, China). Acetate buffer solution (0.1 M HAc-NaAc, pH = 5.0) was used as a supporting electrolyte. All aqueous solutions were prepared with deionized water (DIW).

### 2.2. Characterization

High-resolution transmission electron microscope (TEM, JEOL/JEM-2100, Japan) was used to observe the morphology of rGO-Pd. Powder D8 Advance X-ray diffraction (XRD, Bruker AXS D8) and 3D Nanometer Scale Raman PL Microspectrometer (Tokyo Instruments, Inc., a 785 nm He-Ne laser) were employed to analyze the chemical components of GO, rGO, and rGO-Pd hybrid. The morphology of PDA-Lac-rGO-Pd composite was observed with a Hitachi SU1510 SEM. Fourier transform infrared (FT-IR) spectra were recorded in the range of 500–4000 cm^−1^ on a Nicolet iS10 FT-IR spectrometer (Thermo Fisher Scientific).

### 2.3. Preparation of PDA-Lac-rGO-Pd Composite and Biosensors

**Figure 1:**
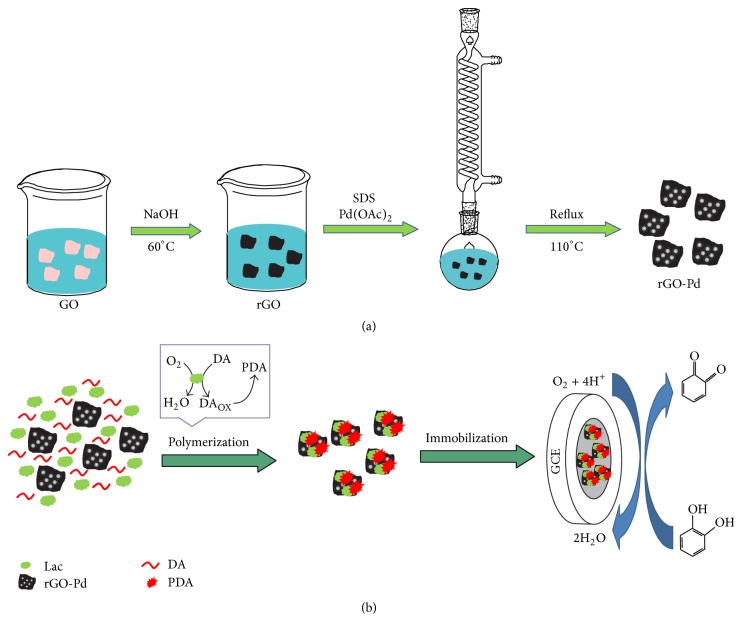
Schematic of the fabrication process of (a) rGO-Pd and (b) PDA-Lac-rGO-Pd composite and modified electrode.

Prior to use, GO was prepared from expanded graphite powder by Hummers' method [22]. rGO-Pd was prepared according to the method reported in literature [23]. The fabrication process is shown in Figure 1(a). To begin with, sodium hydroxide solution was added into the prepared GO suspension with a few minutes of sonication at 60°C to obtain rGO suspension [24]. Next, 20 mL of sodium dodecyl sulfate (SDS, 0.1 mol/L) solution was added into the rGO suspension (2.5 mg/mL) followed by 5 min of sonication. After that, 10 mg of palladium acetate was added into the previous solution, and the mixture was refluxed at 110°C with magnetic stirring for 4 h. Lastly, the mixture was washed by deionized water and centrifuged for several times and dried to obtain the rGO-Pd hybrids. Herein, the SDS decomposed to 1-dodecanol when it was heated, which can reduce Pd(OAc)_2_ to Pd [25].

1 mL rGO-Pd (1.0 mg mL^−1^) suspension was prepared with the aid of sonication for ca. 5 min. Then, 1 mL Lac solution (1.0 mg mL^−1^) and 1.0 mL DA solution (2.8 mg mL^−1^) were successively added in the rGO-Pd suspension under continuous stirring for 3 h. As shown in Figure 1(b), self-polymerization of DA produced polydopamine (PDA), which could wrap the Lac on the surface of rGO-Pd, achieving the immobilization of Lac and the polydopamine functionalization of rGO-Pd. By this way, we obtained the PDA-Lac-rGO-Pd composite.

Before modification, the glass carbon electrode (GCE) was polished to a mirror-like surface with alumina slurry and washed by DIW. Next, the electrode was immersed in ethanol for 5 min with the aid of ultrasonic to remove the adsorbed alumina slurry. After that, the as-prepared electrode was dried in nitrogen. As shown in Figure 1(b), 10 *µ*L of PDA-Lac-rGO-Pd suspension was dropped onto the bare electrode, followed by drying at 4°C in a refrigerator to obtain the PDA-Lac-rGO-Pd/GCE modified electrode. In compared experiments, PDA-Lac/GCE and PDA-Lac-rGO/GCE were prepared by using similar methods, maintaining the equal amounts of DA, Lac, and rGO.

### 2.4. Electrochemical Measurements

Electrochemical experiments were conducted by using a CHI 660E electrochemical workstation (CH Instruments, Inc., Shanghai, China). A three-electrode cell was employed for electrochemical tests, which contained a GCE, a platinum wire auxiliary electrode, and an Ag/AgCl reference electrode. Before electrochemical experiments, the test solution was bubbled with highly pure nitrogen for 20 min to exclude the influence of oxygen except the amperometric analysis.

## 3. Results and Discussion

### 3.1. Characterization

**Figure 2:**
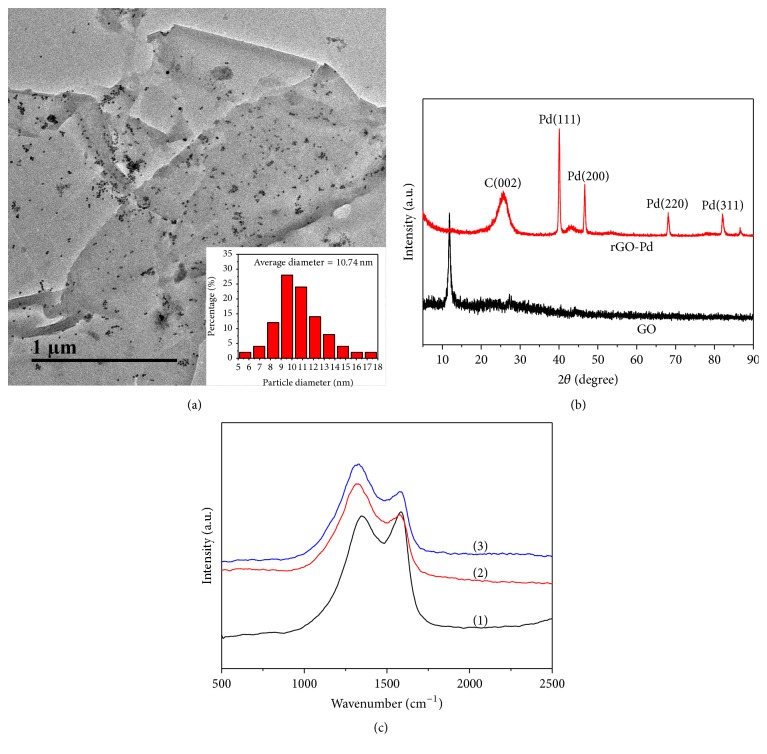
(a) TEM image of rGO-Pd hybrid, inset: diameter distribution of Pd nanoparticles; (b) XRD patterns of GO and rGO-Pd; (c) Raman spectra of (1) GO, (2) rGO, and (3) rGO-Pd hybrid.

Figure 2(a) shows the transmission electron microscopy (TEM) image of rGO-Pd hybrid. It can be seen that the rGO presents a laminated structure with almost transparent color, which is in accordance with the morphologies reported in literatures [26, 27]. Meanwhile, a large number of small nanoparticles were evenly dispersed on the surface of rGO; only very few aggregation particles can be observed. These nanoparticles may be Pd nanoparticles, which are the reduction products of palladium acetate. As shown in the inset of Figure 2(a), the average diameter of the nanoparticles is ca. 11 nm, so small sizes of Pd nanoparticles are favorable to develop their electrocatalytic properties.

X-ray diffraction (XRD) was employed to investigate the chemical components of GO and rGO-Pd. As shown in Figure 2(b), only one diffraction peak at ca. 11.0° can be seen on the curve for GO; this peak is related to a basal spacing of stacked GO. However, for the XRD pattern for rGO-Pd, there are other five relatively obvious diffraction peaks appearing at 25.0°, 40.1°, 46.6°, 68.1°, and 82.1°, which are corresponded to the (002) plane of graphite and the (111), (200), (220), and (311) face-center cubic lattice planes of Pd, respectively [28, 29]. The XRD result suggested that the GO was chemically reduced to rGO, and Pd nanoparticles were generated on the rGO surface. Raman spectroscopy is usually utilized to characterize the structures of carbon materials. Herein, it was employed to investigate the structure changes of GO, rGO, and rGO-Pd; the result is shown in Figure 2(c). For GO, there are two distinct peaks, appearing at around 1346 cm^−1^ and 1590 cm^−1^, which are corresponded to D band and G band, respectively. The D band is relative to the out-of-plane sp^3^ vibrations of carbons, while the G band can be ascribed to the carbon in-plane sp^2^ vibrations [30, 31]. The intensity ratio of the D band and G band (*I*_D_/*I*_G_) reflects the size of sp^2^-carbon domain and the quantity of defect sites. The *I*_D_/*I*_G_ value of GO is 0.96, while this value for rGO and rGO-Pd increases to 1.34 and 1.36, indicating that rGO and rGO-Pd harbor more defects than GO, which may be attributed to the smaller size of the rGO sheets [32]. It is noticeable that the spectra of rGO and rGO-Pd are blue shifted in comparison with that of GO; this can be explained by the damage of single and double carbon bonds within sp^2^ carbon ribbons during the reaction [33]. The Raman spectra indicate the successful reduction of GO. The XRD and Raman spectra characterizations jointly reveal the successful synthesis of rGO-Pd hybrid.

**Figure 3:**
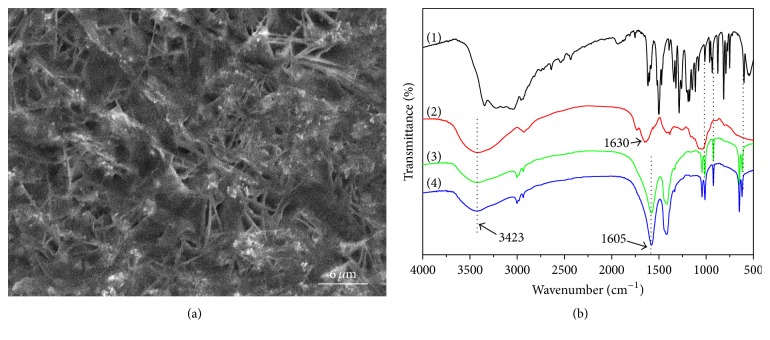
(a) SEM image of the surface of PDA-Lac-rGO-Pd/GCE; (b) FT-IR spectra of (1) DA, (2) Lac, (3) PDA-Lac composite, and (4) PDA-Lac-rGO-Pd composite.

Figure 3(a) displays the scanning electron microscopy (SEM) image of the surface of PDA-Lac-rGO-Pd/GCE. It is manifested that PDA-Lac-rGO-Pd composite forms a porous membrane on the GCE surface. Those “pores” and “ravines” are beneficial to the substrate diffusion. Besides, the “white” nanoparticles on the composite surface may be the excess Pd nanoparticles which fell off from the rGO surface during the synthesis reaction process.  Figure 3(b) shows the FT-IR spectra of DA, Lac, PDA-Lac composite, and PDA-Lac-rGO-Pd composite. The spectrum for DA (curve (1)) is similar to that reported in literature [34]. Those peaks below 1200 cm^−1^ are related to the in-plane bending vibrations of phenolic groups and CH/CH_2_ groups, which also can be found in the spectra of PDA-Lac and PDA-Lac-rGO-Pd composites (spectrum (3) and spectrum (4), resp.) [35]. As shown in spectrum (2), a broad absorption peak at ca. 3423 cm^−1^ is assigned to NH and OH stretching vibrations in the proteins, which also occurs in curve (c) and curve (d) [36]. The peak at ca. 1630 cm^−1^ can be ascribed to the characteristic absorption peak of amide I (1700–1600 cm^−1^), and this peak is shifted to ca. 1605 cm^−1^ in spectra (c) and (d), indicating the strong interaction reaction between Lac and DA [37]. Notably, spectrum (c) and spectrum (d) show almost same absorption peaks, implying that the addition of rGO-Pd does not affect the composite chemical structure. The FT-IR test result demonstrates the successful synthesis of PDA-Lac-rGO-Pd composite.

### 3.2. Electrochemical Studies

**Figure 4:**
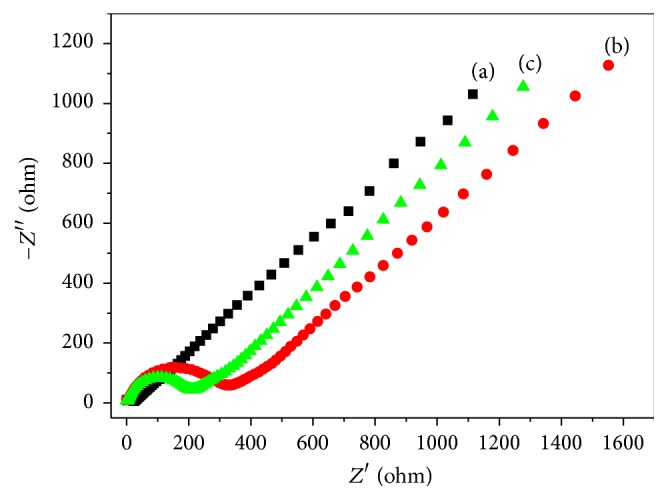
EIS of modified electrodes: (a) bare GCE, (b) PDA-Lac/GCE, and (c) PDA-Lac-rGO-Pd/GCE in 0.1 M KCl containing 5 mM Fe(CN)_6_^3−/4−^. Frequency range: 0.01 Hz–100000 Hz. Amplitude: 5 mV.

Electrochemical impedance spectroscopy (EIS) experiments were conducted to investigate the interface resistances of modified electrodes. As shown in Figure 4, the semicircle parts of curves are corresponding to the electron transfer processes occurring on the modified electrodes and the electron transfer resistance of the electrochemical reaction (Ret) is related to the semicircle diameter. Obviously, bare GCE (curve (a)) shows a small semicircle, which almost cannot be observed, implying the negligible interface resistance of bare GCE. However, after PDA-Lac composite was immobilized on the GCE, the semicircle diameter (curve (b)) was apparently increased; the Ret value is around 330 Ω. This demonstrates that the interface resistance of PDA-Lac/GCE is increased in comparison with that of bare GCE because of the poor conductivity of PDA-Lac composite. While, for the PDA-Lac-rGO-Pd/GCE, the semicircle diameter (curve (c)) is decreased to some extent, the Ret value is reduced to about 200 Ω. This phenomenon can be explained by the fact that the electronic conduction ability of PDA-Lac-rGO-Pd composite was improved by the addition of rGO-Pd with good conductivity.

**Figure 5:**
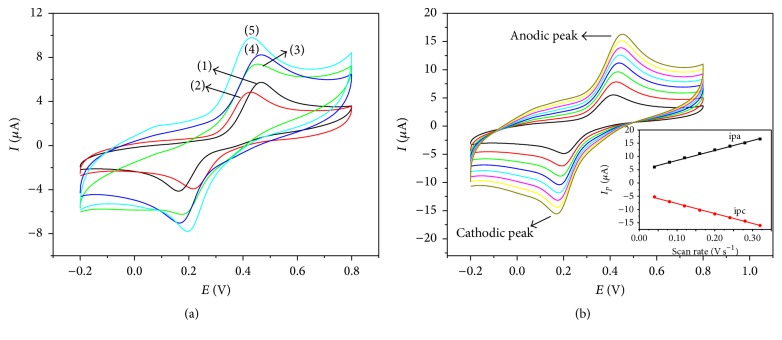
(a) Cyclic voltammograms of (1) bare GCE, (2) PDA/GCE, (3) PDA-Lac/GCE, (4) PDA-Lac-rGO/GCE, and (5) PDA-Lac-rGO-Pd/GCE in 0.1 M acetate buffer solution (pH 5.0) containing 220 *µ*M catechol at 100 mV s^−1^. (b) Cyclic voltammograms of PDA-Lac-rGO-Pd/GCE in 0.1 M acetate buffer solution (pH 5.0) containing 220 *µ*M catechol at scan rates of 40, 80, 120, 160, 200, 240, 280, and 320 mV s^−1^ (from inner to outer), respectively. Inset: plots of the corresponding anodic and cathodic peak currents versus scan rate.

Figure 5(a) compares the electrocatalytic performances of different electrodes toward 220 *µ*M catechol in 0.1 M pH 5.0 acetate buffer solution by using cyclic voltammetry. Bare GCE (curve (1)) shows a pair of redox peaks, which are located at around 0.47 V and 0.16 V, respectively, indicating the electrochemical redox reaction of catechol on the surface of bare GCE. Compared with bare GCE, the redox peaks of PDA/GCE (curve (2)) are decreased to some extent, which may be caused by the inhibiting effect of PDA layer toward electron transfer. Nevertheless, due to high-efficient catalysis of Lac toward catechol, not only the anodic peak but also the cathodic peak of PDA-Lac/GCE (curve (3)) possesses higher current value than those of bare GCE and PDA/GCE. It is noticeable that the anodic peak current and cathodic peak current of PDA-Lac-rGO/GCE (curve (4)) are increased by around 0.83 *µ*A and 0.77 *µ*A as compared to those of PDA-Lac/GCE, respectively. This demonstrates that addition of rGO accelerates the electron transfer of electrode, further leading to higher response sensitivity of modified electrode to catechol. A pair of redox peaks are observed on PDA-Lac-rGO-Pd/GCE (curve (5)); the anodic peak and cathodic peak are located at about 0.43 V and 0.20 V, respectively. In addition, the anodic peak current and cathodic peak current are 9.78 *µ*A and −7.79 *µ*A, respectively, which are higher than those of PDA-Lac-rGO/GCE, suggesting the higher sensitivity of PDA-Lac-rGO-Pd/GCE. This can be attributed to the larger number of electroactive sites of rGO-Pd offered by Pd nanoparticles and synergetic catalysis of Pd nanoparticles. The whole electrochemical reaction is a quasi-reversible cyclic process and the sensing mechanism of Lac on PDA-Lac-rGO-Pd/GCE is illustrated in Figure 1(b). Under the presence of molecular oxygen, catechol is oxidized to 1,2-benzoquinone by Lac, coupled with the electrocatalytic reduction of oxygen to water on the surface of GCE. The reaction process can be described as follows:

(1) catechol+Lacoxy⟶1,2-benzoquinone+Lacdeoxy+2H++2e−

(2) Lacdeoxy+O2+4H+⟶Lacoxy+2H2O

Figure 5(b) shows influence of scan rate on cyclic voltammograms of PDA-Lac-rGO-Pd/GCE in 0.1 M acetate buffer solution (pH 5.0) containing 220 *µ*M catechol. The oxidation peaks (anodic peaks) and reduction peaks (cathodic peaks) simultaneously increase with the increment of scan rates. As shown in the inset of Figure 5(b), both of oxidation peak currents and reduction peak currents grow linearly with the scan rate and are proportional to the scan rates. It means that the electrochemical reaction is a surface-controlled process at PDA-Lac-rGO-Pd/GCE.

### 3.3. Condition Optimization

**Figure 6:**
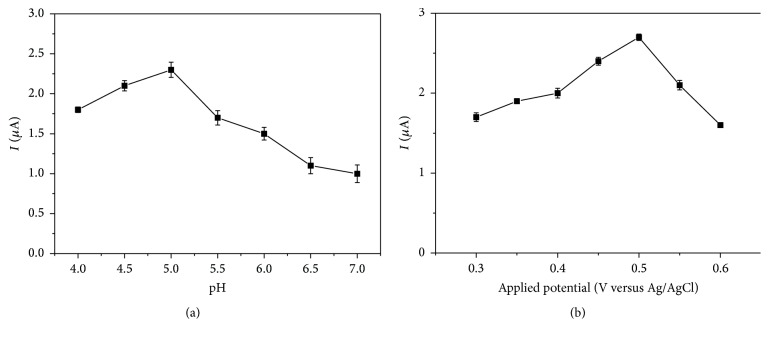
(a) The pH effects and (b) applied potentials of PDA-Lac-rGO-Pb/GCE on the catalytic currents of catechol in 0.1 M acetate buffer solution containing 100 *µ*M catechol.

To acquire the optimum analytic performance, the test environment including solution pH and applied work potentials was optimized before the chronoamperometry experiment, and the result is shown in Figure 6. Obviously, the highest catalytic response currents happen when the solution pH is 5.0 and applied potential is 0.5 V, respectively. So, in the following experiments, these two parameter values were chosen as the fixed values.

### 3.4. Amperometric Analysis of Catechol

**Figure 7:**
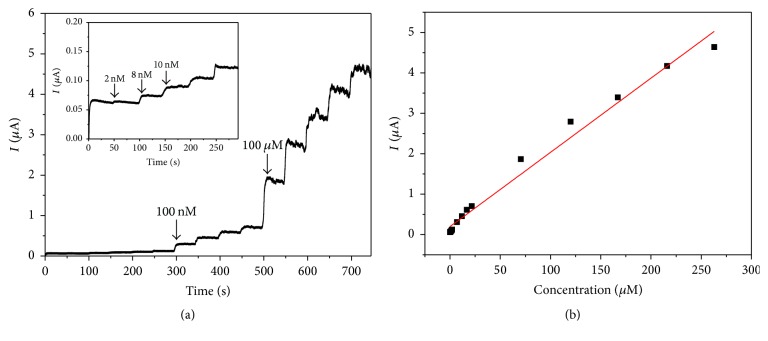
(a) Chronoamperometric response of PDA-Lac-rGO-Pd/GCE on successive addition of different concentration and volume of catechol solutions into pH 5.0 0.1 M acetate buffer solution; applied potential: 0.5 V. Inset: a magnification of the line before 300 s; (b) the calibration curve of PDA-Lac-rGO-Pd/GCE for catechol.

The steady-state amperometric responses of the PDA-Lac-rGO-Pd/GCE to different concentration of catechol were determined by the successive addition of different volumes of 0.2 mM, 2 mM, and 20 mM catechol into 20 mL pH 5.0 acetate buffer solution with stirring under the optimum conditions; the result is shown in Figure 7(a). It can be seen from the inset of Figure 7(a) that the first current step occurred when 2 nM of catechol was added into acetate buffer solution, indicating high sensitivity and low detection limit of PDA-Lac-rGO-Pd/GCE toward catechol. It is noticeable that the step current can reach 95% of its maximum value within 5 s, indicating the fast response of biosensor, which can be explained by the fact that the porous structure of PDA-Lac-rGO-Pd composite is favorable to the fast transfer of electrons. With the successive addition of catechol into solution, the steady-state current values gradually increased.  Figure 7(b) shows the calibration curve of response currents versus substrate concentrations; the response currents increase linearly with the catechol concentrations. The linear range is from 0.1 *µ*M to 263 *µ*M with a correlation coefficient (*R* ^2^) of 0.983 (*n* = 15). The sensitivity is 18.4 *µ*A mM^−1^ and the detection limit is estimated to be 0.03 *µ*M at a signal-to-noise ratio of 3 (S/N = 3).  Table 1 shows biosensing performance comparison of different Laccase modified electrodes toward catechol. It can be clearly seen that the PDA-Lac-rGO-Pd/GCE possesses satisfactory biosensing property toward catechol with low detection limit, high sensitivity, and wide linear range.

### 3.5. Selectivity and Stability of Biosensor

**Figure 8:**
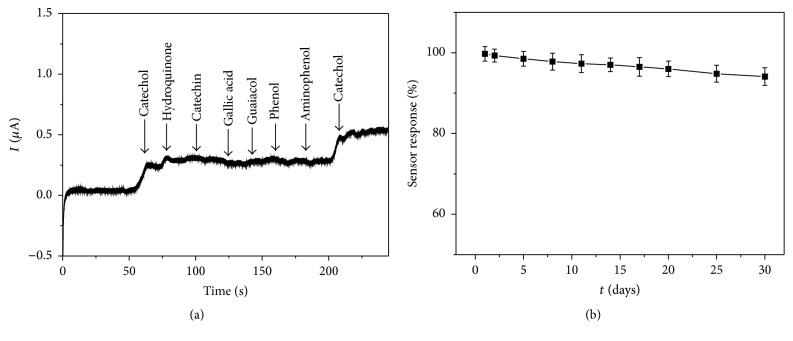
(a) Amperometric responses of PDA-Lac-rGO-Pb/GCE after the addition of different interfering substances; (b) long-term storage stability of PDA-Lac-rGO-Pb/GCE in 0.1 M acetate buffer solution (pH 5.0) at 4°C.

The repeatability, reproducibility, anti-interference, and stability of PDA-Lac-rGO-Pd/GCE were also investigated. The relative standard deviation (RSD) value for 8 successive measurements is 1.6%, implying good repeatability. To study the reproducibility of biosensor, five PDA-Lac-rGO-Pd/GCE were, respectively, prepared under the same condition and the RSD value is calculated to be 3.2%, which demonstrated that the PDA-Lac-rGO-Pd/GCE possessed acceptable reproducibility.  Figure 8(a) shows the catalytic response current of PDA-Lac-rGO-Pd/GCE toward different substances with same concentrations (6 *µ*M), including catechol, hydroquinone, catechin, gallic acid, guaiacol, phenol, and aminophenol. No obvious interference of these interferences to catechol detection can be observed, indicating the anti-interference of PDA-Lac-rGO-Pd/GCE.  Figure 8(b) displays the long-term storage stability of PDA-Lac-rGO-Pd/GCE in 0.1 M acetate buffer solution (pH 5.0) at 4°C. Through a long-term (30 days) storage, the current response value of PDA-Lac-rGO-Pd/GCE still maintained 94.1% of initial value, indicating the excellent stability of PDA-Lac-rGO-Pd/GCE, which may be attributed to the fact that the activity of Lac was well maintained by the biocompatible microenvironment provided by the PDA-rGO-Pd matrix. These results demonstrate that the PDA-Lac-rGO-Pd/GCE possesses good repeatability, reproducibility, anti-interference, and stability.

### 3.6. Real Sample Analysis

The practical application of the PDA-Lac-rGO-Pd/GCE biosensor was also investigated by using untreated water from Taihu Lake, Wuxi, China. Herein, recovery experiment was used and repeated for five times. The analytical results are shown in Table 2. The recovery values for five tests are 104.5%, 98.5%, 101%, 96.5%, and 98%, respectively, and the RSD for these values is only 3.14%, demonstrating the successful application of the novel PDA-Lac-rGO-Pd/GCE biosensor in real water sample analysis.

## 4. Conclusions

In summary, rGO-Pd hybrid was facilely synthesized by a simple method. PDA-Lac-rGO-Pd composite was prepared by a one-pot Lac-catalyzed oxidation of DA in an aqueous suspension containing Lac, rGO-Pd, and DA. A novel biosensor based on the PDA-Lac-rGO-Pd composite was fabricated. Consequently, the biosensor showed excellent biological electrocatalysis toward phenolic pollutant, catechol, with low detection limit, high sensitivity, and wide linear range, as well as good repeatability, reproducibility, anti-interference, and stability. The biosensor was also successfully applied in catechol detection in lake water. This novel composite based on rGO further expands the application of graphene materials in the field of biosensing and paves the way in developing highly sensitive phenolic biosensor.

## Figures and Tables

**Table 1:** Sensing performance comparison of different modified electrodes toward catechol^a^.

Electrodes	Detection limit (*µ*M)	Linear range (*µ*M)	Sensitivity (*µ*A mM^−1^)	Reference
GCE/MCN/Tyr	0.01	0.05–12.5	593.1	[38]
Lac/AP-rGOs/Chit/GCE	7	15–700	15.79	[5]
MB-MCM-41/PVA/lac	0.331	4–87.98	—	[39]
Cu-OMC/Lac/CS/Au	0.67	0.67–13.8	104	[40]
Lac-FSM7.0-GC	2	2–100	—	[41]
Cu/CNFs/Lac/Nafion/GCE	1.18	9.5–9760	33.1	[42]
N-GCE	0.2	5–260	—	[43]
Tyr-AuNPs-DHP/GCE	0.17	2.5–95	115	[44]
PDA-Lac-rGO-Pb/GCE	0.03	0.1–263	18.4	This work

^a^The dashes in the table represent values that were not reported in the references.

**Table 2:** Determination of catechol content in real water samples (*n* = 5).

Sample^a^	C_added_ (*µ*M)	C_found_ (*µ*M)	Recovery (%)	RSD (%)
1	2.00	2.09	104.50	3.14
		1.97	98.50	
		2.02	101.00	
		1.93	96.50	
		1.96	98.00	

^a^1: Taihu Lake water.
